# Pomegranate seed as a novel source of plant protein: Optimization of protein extraction and evaluation of in vitro digestibility, functional, and thermal properties

**DOI:** 10.1002/fsn3.4242

**Published:** 2024-06-05

**Authors:** Souri Oroumei, Karamatollah Rezaei, Hooman Chodar Moghadas

**Affiliations:** ^1^ Department of Food Science, Engineering, and Technology University of Tehran Karaj Iran

**Keywords:** essential amino acids, functional properties, Plackett–Burman design, protein isolate, response surface methodology

## Abstract

This research was carried out to optimize the extraction process of proteins from pomegranate seeds and characterize their in vitro digestibility as well as their thermal and functional properties. For this purpose, the study screened five parameters (liquid/solid ratio, pH, temperature, NaCl concentration, and time) that could potentially influence the extraction process. This screening was conducted using a two‐level Placket–Burman design (PBD). The significant parameters (pH and NaCl concentration) were subsequently optimized using a three‐level face‐centered central composite design (FCCD) to determine the optimum extraction conditions. A maximum protein recovery of 83.8% was obtained at pH 11.0 and NaCl concentration of 0.0 M. Pomegranate seed protein isolate (PSPI) with a protein content of 92.4% (w/w) was obtained through the isoelectric precipitation of pomegranate seed protein extracted under the optimized conditions. An emulsifying activity index of 14.1 m^2^ g^−1^ was observed at the isoelectric pH, where the emulsion stability index was at 8.2%. PSPI also showed high water‐ and oil‐holding capacities (3.7 and 4.3 g g^−1^, respectively). The essential amino acid levels in PSPI (except for valine and isoleucine) exceeded the recommended amounts set by WHO/FAO/UNU for adults, highlighting its high nutritional value. Based on thermal analysis data, denaturation of PSPI could occur at 89.5°C. The in vitro digestibility of PSPI was found to be 74.3%. PSPI shows a potential as a novel ingredient for substituting animal‐based proteins in various food applications.

## Graphical abstract

The extraction conditions to obtain protein from pomegranate seeds were optimized, and an isolate with a protein content of 92.4% (w/w) was collected. This protein isolate was then analyzed for in vitro digestibility, functional and thermal properties. The results show a potential for the use of pomegranate seed protein isolate as a novel ingredient in food industry.

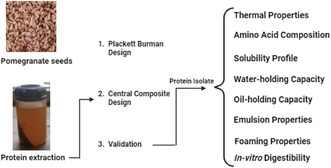

## 1 INTRODUCTION

Proteins are key components of human diet due to their favorable functional and nutritional properties. Animal proteins have long been recognized as the primary protein source for humans. The global demand for meat has experienced substantial growth in recent years, driven by factors such as population growth and economic development. However, concerns have emerged regarding the inefficiencies of meat production and the potential negative impacts of meat consumption on human health (Zhang et al., 2022). As a result, it is crucial to substitute animal‐based proteins with new sources of plant proteins, among which under‐utilized by‐products of the food industry for protein extraction can be considered. This approach not only provides a low‐cost protein source but also can help reduce environmental pollution due to such materials.

As a by‐product of the pomegranate juice industry, pomegranate seed contains numerous functional ingredients such as oil, protein, and fiber (Ko et al., 2021). The main product derived from pomegranate seed is oil, which can primarily be used for human consumption or in cosmetic products (Paul & Radhakrishnan, 2020). López‐Marcos et al. (2015) investigated the dietary fibers extracted from pomegranate seeds for possible applications in food formulations. The results of their study showed that pomegranate seed dietary fiber has promising techno‐functional properties and can be used in supplementary high‐fiber food products. Talekar et al. (2018) investigated the protein hydrolysates of pomegranate seeds obtained after simultaneous enzymatic oil and protein extraction and reported that essential amino acid levels in the protein hydrolysates were beyond the required levels recommended by WHO/FAO/UNU Expert Consultation (2007) for individuals above 3 years old. Considering these findings, pomegranate seed protein (PSP) can be a potential ingredient to be incorporated into food formulations.

Several major extraction procedures including alkaline extraction (Gao et al., 2020), salt‐ (Sun et al., 2022) and enzyme‐assisted extraction (Akyüz & Ersus, 2021), and reverse micelle extraction (Fang et al., 2023) processes have been reported for the recovery of plant proteins. Among them, alkaline extraction stands out as the most promising technique owing to its ease of implementation and cost efficiency (Perović et al., 2020). Some novel extraction techniques such as ultrasound‐ (Rahman & Lamsal, 2021), microwave‐ (Peñas et al., 2023), and pulsed electric field (Zhang et al., 2021) extraction have also been developed that can be examined for the extraction of proteins from different sources. During protein extraction from plant sources, various parameters including pH, temperature, liquid/solid ratio, ionic strength, and time can impact the extractability of proteins (Feyzi et al., 2018; Garg et al., 2020). However, there has been no investigation into how extraction conditions affect PSP recovery. Additionally, as a novel plant‐based protein source, PSP has not yet been explored for its functional properties, which was considered in the current study as the extraction process was optimized. A Plackett–Burman design (PBD) from Design‐Expert™ was initially implemented to determine factors impacting the PSP extraction. Afterward, the factors identified as significant for protein extraction were further optimized by Face‐Centered Central Composite Design (FCCD) from response surface methodology (RSM) to get a higher yield. The “one‐factor‐at‐a‐time” way of experiments is limited in its ability to evaluate interactions among independent variables and provide comprehensive information on the effects of all variables (Zhou et al., 2018). PBD is frequently used to screen significant factors among multiple variables, allowing to identify main independent variables influencing the extraction process. These significant factors are then optimized using RSM, which helps determine the relationships among the variables and responses. The combination of PBD and RSM, known as PBD‐RSM, overcomes the limitations of the classic “one‐factor‐at‐a‐time” approach (Zhou et al., 2015). PBD‐RSM enables the evaluation of multiple parameters and their interactions based on quantitative data, providing a deeper insight into the extraction process. The study further investigated the functional properties, thermal characteristics, and in vitro digestibility of pomegranate seed protein isolate (PSPI) under optimized conditions.

## 2 MATERIALS AND METHODS

### 2.1 Materials

Seeds from the Malas‐e‐Torsh‐e‐Saveh variety of pomegranate (*Punica granatum* L.) were acquired from a local juice‐producing company located in Saveh, Markazi Province, Iran. The seeds were transferred to the laboratory and air‐dried under ambient conditions (25 ± 5°C) until they reached a constant weight. The dried seeds were refrigerated at 4°C until analysis. Analytical‐grade chemicals from Merck Chemical Co. (Darmstadt, Germany) were used in this study.

### 2.2 Preparation of defatted pomegranate seed meal (DPSM)

For the preparation of DPSM, pomegranate seeds were first ground and then sieved using a 40‐mesh sieve and subsequently defatted for 6 h using a Soxhlet apparatus applying hexane as solvent at four times volume to weight ratio. The DPSM was then air‐dried at ambient conditions (25 ± 5°C) for 24 h and stored at 4°C in sealed bags until they were used for further analysis.

### 2.3 Extraction procedures

#### 2.3.1 Extraction of pomegranate seed protein (PSP)

To obtain PSP, 10.0 g of DPSM (with 9.9% (w/w) moisture, 23.5% protein, 0.6% fat, 44.3% carbohydrate, 18.8% fiber, and 2.7% ash) was mixed with NaCl solution (0.0–1.0 M) in a 300‐mL beaker at a specific ratio of 20:1 to 10:1 (v/w). The pH was then adjusted within the 9.0–11.0 range (as needed) using a 1.0‐M NaOH solution. The suspension was then shaken for 1–2 h at 25–40°C followed by centrifugation at 10,000 *g* for 30 min at 4°C using a Sigma 8KS refrigerated centrifuge (Germany). The soluble protein content from the above stage was quantified using the Kjeldahl procedure, applying a nitrogen‐to‐protein conversion factor of 6.25 (AOAC, 1990). Equation (1) was used to determine the protein yield:

(1) $$Y\;\left(\%\right)=\frac{C_{s}V}{m}\times 100$$

where *Y* is the protein yield, *C*_*s*_ is the soluble protein concentration in the DPSM solution (mg L^−1^), *V* represents the volume of the DPSM solution (L), and *m* denotes the protein content (mg) of the DPSM. To prepare PSPI, the supernatant obtained under the optimum conditions from the RSM experiments was acidified using 0.5‐M HCl solution until it reached pH 5.0, which is the isoelectric pH of PSP. To precipitate proteins, the mixture was then centrifuged at 10,000 *g* (Sigma 8KS centrifuge system) for 30 min at 4°C. The resulting precipitate was rinsed twice with distilled water and then centrifuged again for 10 min at 10,000 *g*. After dissolving in distilled water and pH adjustment to 7.0 (using 0.5‐M NaOH), the solution was freeze‐dried and kept under refrigerated conditions until analysis.

#### 2.3.2 Selection of significant parameters for extracting PSP using Plackett–Burman design (PBD)

In this study, a two‐level PBD with 12 experiments was used to identify the significant parameters influencing PSP extraction. The pH (*A*), NaCl concentration (*B*), temperature (*C*), liquid/solid ratio (*D*), and time (*E*) at two levels each were evaluated (Table 1). The other variables in the design (*X*_6_–*X*_11_) were some dummy factors introduced by the software, which were applied to estimate the experimental errors during the data analysis (Sha et al., 2019). The obtained responses were correlated with the independent variables using the first‐order polynomial model in Equation (2):

(2) $$Y=\beta_{0}+\sum_{i=1}^{5}\beta_{i}X_{i}$$

**TABLE 1:** Plackett–Burman design from Design Expert™ with coded and actual experimental values (in brackets) and test results (protein yield) for screening significant variables for the extraction of protein from pomegranate seeds.

Experimental variables^a^						Observed values
Runs	*A* (pH)	*B* (M)	*C* (°C)	*D* (mL g^−1^)	*E* (h)	Protein yield (%, w/w)
1	1 (11)	1 (1)	−1 (25)	−1 (10:1)	−1 (1)	27.8
2	1 (11)	−1 (0)	1 (40)	1 (20:1)	−1 (1)	79.8
3	−1 (9)	−1 (0)	1 (40)	1 (20:1)	1 (2)	78.9
4	−1 (9)	−1 (0)	−1 (25)	−1 (10:1)	−1 (1)	61.0
5	1 (11)	1 (1)	−1 (25)	1 (20:1)	1 (2)	24.2
6	1 (11)	−1 (0)	−1 (25)	1 (20:1)	1 (2)	71.7
7	1 (11)	1 (1)	1 (40)	−1 (10:1)	1 (2)	31.4
8	−1 (9)	1 (1)	−1 (25)	1 (20:1)	−1 (1)	18.8
9	−1 (9)	−1 (0)	−1 (25)	−1 (10:1)	1 (2)	54.3
10	−1 (9)	1 (1)	1 (40)	1 (20:1)	−1 (1)	17.9
11	1 (11)	−1 (0)	1 (40)	−1 (10:1)	−1 (1)	71.7
12	−1 (9)	1 (1)	1 (40)	−1 (1:20)	1 (2)	15.7

^a^ *A*: pH of the extraction, *B*: NaCl concentration (M), *C*: extraction temperature (°C), *D*: liquid/solid ratio (mL g^−1^), *E*: extraction time (h).

In the equation, *Y* represents the predicted response, $X_{i}$ denotes an independent variable, $\beta_{0}$ is the model intercept and $\beta_{i}$ is linear regression coefficient. The selection of the variables and their corresponding experimental values were based on both preliminary experiments conducted in this study and several reports from the literature. A Pareto chart was investigated to determine the significance of each variable relative to the response. Variables with significance levels (*p* < .05) were selected for the optimization by FCCD using RSM in the next section.

#### 2.3.3 Extraction optimization using response surface methodology

After identifying the significant variables from the previous stage (PBD), the extraction conditions of PSP were optimized by FCCD of RSM with two independent variables at three levels. Effects of pH (*A*) and NaCl concentration (*B*) as independent variables were investigated on the extraction yield (*Y*) of PSP at a given set of other conditions (25°C temperature, liquid/solid ratio of 20:1, v/w, over 60‐min time) was studied. The experimental conditions (13 runs with five runs at the central point) along with the coded values for each independent variable, are shown in Table 2. A multiple regression analysis was carried out by applying the quadratic polynomial model presented in Equation 3 (Myers et al., 2016):

(3) $$Y=\beta_{0}+\sum_{i=1}^{n}\beta_{j}X_{j}+\sum_{i=1}^{n}\beta_{\mathrm{jj}}X_{j}^{2}+\sum \sum_{i<j}\beta_{\mathrm{ij}}X_{i}X_{j}\left(n=2\right)$$

**TABLE 2:** Face‐centered central composite design from response surface methodology (RSM) with actual experimental values (in brackets), coded values, and test results for the optimization of conditions in the protein extraction from pomegranate seed.

Runs	Experimental variables^a^		Observed values
	*A* (pH)	*B* (M)	Protein yield (%, w/w)
1	−1 (9)	−1 (0)	75.3
2	1 (11)	0 (0.5)	57.3
3	0 (10)	0 (0.5)	49.3
4	−1 (9)	0 (0.5)	29.4
5	−1 (9)	1 (1)	16.4
6	1 (11)	1 (1)	28.6
7	1 (11)	−1 (0)	83.4
8	0 (10)	1 (1)	20.5
9	0 (10)	−1 (0)	79.1
10	0 (10)	0 (0.5)	43.8
11	0 (10)	0 (0.5)	43.0
12	0 (10)	0 (0.5)	42.4
13	0 (10)	0 (0.5)	41.8

^a^ *A*: Extraction pH, *B*: NaCl concentration (M).

In the equation, *Y* represents the predicted response, $X_{i}$ and $X_{j}$ denote the coded independent parameters, n is the number of variables, $\beta_{0}$ is the intercept and $\beta_{j}$, $\beta_{\mathrm{jj}}$, and $\beta_{\mathrm{ij}}$ represent the regression coefficients of the linear, square, and interaction terms, respectively.

### 2.4 Determination of amino acid composition

A method by Hayati Zeidanloo et al. (2019) was applied to determine the amino acid composition of PSPI. In brief, PSPI samples (200 mg each) were treated with 10 mL 6.0 N HCl solution (110°C for 24 h) in sealed tubes under a nitrogen atmosphere, and then the treated samples were filtered using a 0.45 μm membrane filter. Afterward, the samples were dried in a vacuum oven at 40°C and then subjected to derivatization using a solution consisting of ethanol‐water‐triethylamine‐phenylisothiocyanate in a 7:1:1:1 (v/v/v/v) ratio. This process was carried out for 20 min at 25°C under a nitrogen atmosphere. The amino acid composition was then determined using an HPLC system (Waters, Milford, MA) equipped with a dual‐absorbance UV detector at 254 nm (Model 2487, Waters) and Pico‐Tag column. Mobile phase was a mixture of acetonitrile:water at 6:4 ratio (v/v) flowing at 1 mL min^−1^. Amino acids were identified according to their corresponding retention times in a mixture of standard amino acids, and their quantification was estimated using the relative peak areas of amino acids.

### 2.5 Thermal properties

A differential scanning calorimeter system (TA Instruments DSC‐TGA model SDT‐Q600 simultaneous analyzer, New Castle, DE) was used for the thermal analysis of PSPI, according to Feyzi et al. (2018). After weighing 40.0 mg PSPI sample and sealing the aluminum pan, it was heated from 40 to 200°C at a rate of 2°C min^−1^. Peak denaturation temperature (*T*_*d*_) and enthalpy of denaturation (Δ*H*) were determined using an empty pan serving as the reference.

### 2.6 Functional properties

#### 2.6.1 Protein solubility profile

Assessment of PSPI solubility was performed as described by Gundogan and Karaca (2020) with slight modifications. First, 0.5 g sample was dispersed in 50 mL water, and using either NaOH (1.0 M) or HCl (1.0 M) solution, the pH of the mixture was adjusted from 2.0 to 11.0. After stirring the mixture for 2 h at room temperature on a magnetic stirrer, it was centrifuged for 15 min at 8000 *g* (Universal 320, Hettich Zentrifugen, Germany). Kjeldahl procedure was employed to determine the protein content of each supernatant using a conversion factor of 6.25. Finally, protein solubility was determined according to Equation (4):

(4) $$\text{Protein}\;\text{solubility}\;\left(\%\right)=\frac{\text{The}\;\text{content}\;\text{of}\;\text{protein}\;\text{in}\;\text{the}\;\text{collected}\;\text{supernatant}}{\text{The}\;\text{content}\;\text{of}\;\text{protein}\;\text{in}\;\text{PSPI}}\times 100$$

#### 2.6.2 Water‐holding capacity

Water‐holding capacity (WHC) of PSPI was determined according to Garg et al. (2020) as follows. Briefly, 0.2 g of each sample was dispersed in 10 mL distilled water, and after thorough mixing (for 2 min) with a vortex, it was allowed to stay at ambient conditions for 1 h before it was centrifuged at 4000 *g* for 15 min (Universal 320 centrifuge from Hettich Zentrifugen, Germany). The tube was then weighed after discarding the supernatant and WHC was determined following Equation (5):

(5) $$\mathrm{WHC}\;{\left(g\;g\right)}^{-1}=\left(W_{2}-W_{1}\right)/W_{0}\times 100$$

In the equation, *W*_0_ represents the weight of the dry sample, *W*_1_ denotes the combined weight of the test tube and dry sample and *W*_2_ is the weight of the test tube containing precipitate after soaked in water.

#### 2.6.3 Oil‐holding capacity

The oil‐holding capacity (OHC) of PSPI was carried out following a procedure described by Garg et al. (2020). A 0.2‐g sample was dispersed in 10.0 mL sunflower oil in a pre‐weighed test tube and mixed using a vortex for 2.0 min and then centrifuged for 1 h at 4000 *g* for 15 min (Universal 320, Hettich Zentrifugen). After discarding the supernatant, the test tube containing the precipitate was weighed. OHC was determined using Equation (6):

(6) $$\mathrm{OHC}\;{\left(g\;g\right)}^{-1}=\left(W_{2}-W_{1}\right)/W_{0}\times 100$$

where *W*_0_ is the dry sample weight and “*W*_2_ − *W*_1_” is the amount of oil absorbed.

#### 2.6.4 Emulsifying properties

Procedures described by Gundogan and Karaca (2020) were used to determine both the emulsifying activity index (EAI) and the emulsion stability index (ESI). PSPI dispersions were prepared in distilled water at a concentration of 1% (w/v) with pH adjustments made to achieve the desired levels (2.0–11.0) using 1.0 M NaOH or HCl solution. Then, 5.0 mL sunflower oil was mixed with 15.0 mL of each dispersion and homogenized for 2 min at 15,000 rpm using a homogenizer (Ultra‐Turrax T25, IKA, Staufen, Germany). Fifty microliters of the homogenized emulsion was placed in a Falcone tube and 5.0 mL sodium dodecyl sulfate solution (0.1%, w/v) was added, and absorbances were recorded at 500 nm using a UV–Visible spectrophotometer (Model 500DB, Spectrum Instruments, Germany) at time zero and after 10 min of homogenization. Equation (7) was applied to determine the EAI of PSPI:

(7) $$\mathrm{EAI}\;\left(m^{2}\;g^{-1}\right)=\frac{2.303\times 2\times A_{0}\times \text{dilution}\;\text{factor}}{\varphi \times C\times 1000}$$

In the equation, *A*_0_ represents the absorbance measured immediately following homogenization (i.e., at time 0), and *C* refers to the concentration of the dispersion in g mL^−1^. The dilution factor is equal to 100 and *φ* is the volumetric fraction of oil (0.25). ESI was determined using the following expression in relation to the EAI, which was determined at 0.0 and 10.0 min after preparing the emulsion:

(8) $$\mathrm{ESI}\;\left(\%\right)=\frac{{\mathrm{EAI}}^{10}}{{\mathrm{EAI}}^{0}}\times 100$$

#### 2.6.5 Foaming properties

The procedures given by Zhan et al. (2019) were used to determine the foaming capacity (FC) and foam stability (FS) of PSPI. PSPI sample (0.5 g) was dispersed in 50 mL distilled water and then pH levels were adjusted as needed within 2.0–11.0 levels. The mixtures were then homogenized for 2 min at 15,000 rpm using a T25 Ultra‐Turrax homogenizer (IKA, Staufen, Germany). For each mixture, the foam volume was recorded at 0 time and after 30 min of homogenization. FC and FS were determined using the Equations (9) and (10), respectively:

(9) $$\mathrm{FC}\;\left(\%\right)=\left[\left(V_{2}-V_{1}\right)/V_{1}\right]\times 100$$

(10) $$\mathrm{FS}\;\left(\%\right)=\left[\left(V_{2}-V_{3}\right)/\left(V_{2}-V_{1}\right)\right]\times 100$$

where *V*_1_ represents the volume of the prepared mixture before the homogenization, *V*_2_ signifies the volume immediately after the homogenization (0 min), and *V*_3_ denotes the volume at a specified time (30 min).

### 2.7 Digestibility

The digestibility of PSPI under in vitro conditions was evaluated following the method described by Yu et al. (2017). The procedure involved mixing each sample (0.5 g) with 20.0 mL of pepsin dispersion (0.5 mg mL^−1^ in 0.1 M HCl) and shaken for 1.5 h at 37°C in a water bath. Then, using 0.5 M NaOH solution, the dispersion was neutralized and mixed with 10 mL pancreatin solution (2.0 mg mL^−1^ in 0.1 M phosphate buffer at pH 8.0). After incubating the suspension in a water bath (24 h at 37°C), the mixture was placed in boiling water for 5 min to stop the digestion. The mixture was then centrifuged for 20 min at 4400 *g* (Universal 320, Hettich Zentrifugen, Germany) to precipitate undigested solids, which were then air‐dried, weighed, and analyzed for protein content using the Kjeldahl method. Equation (11) was applied to determine protein digestibility:

(11) $$\text{Protein}\;\text{digestibility}\;\left(\%\right)=\left[\left(N_{0}-N_{1}\right)/N_{0}\right]\times 100$$

where *N*_1_ was total protein content in the undigested sample and *N*_0_ was that in PSPI.

### 2.8 Statistical analysis

Design Expert, version 12.0.3, developed by Stat‐Ease Inc. (Minneapolis, MN), was applied to determine the conditions of the experiments according to the PBD and FCCD and to analyze the data from the experiments. The reported tests for the functional properties and in vitro digestibility were all carried out in triplicates, and the data are given as the mean ± SD.

## 3 RESULTS AND DISCUSSION

### 3.1 Screening experimental parameters applied for the protein extraction

The PBD matrix, along with the coded and experimental values for the extraction of protein from pomegranate seeds are summarized in Table 1. Depending on the extraction conditions, the yield of the protein extraction varied over 15.7%–79.8% (w/w). Table 3 presents the data for the statistical analysis of the PBD, demonstrating the significance (*p* < .0001) of the model. With an *R* ^2^ value of .9714, the model demonstrated a good fit with the experimental data. Additionally, the values of predicted *R* ^2^ (.8858) and adjusted *R* ^2^ (.9476) were found to be close to the value of *R* ^2^, suggesting that the predicted and experimental data are in good agreement. Furthermore, a relatively low value of coefficient of variation (C.V. = 12.79%) indicated the reliability and reproducibility of the experiments. The significance levels of the test parameters, as determined by the analysis of variance (ANOVA), followed the order of *B* > *A* > *C* > *D* > *E*.

**TABLE 3:** Analysis of variance (ANOVA) for the yields obtained for the protein extraction from pomegranate seeds using Plackett–Burman design.

Source^a^	Sum of squares	*df* ^b^	Mean square	*F*‐value	*p*‐Value	Significance
Model	7098.11	5	1419.62	40.82	.0001	S
*A*	300.00	1	300.00	8.63	.0260	S
*B*	6608.21	1	6608.21	190.01	<.0001	S
*C*	117.81	1	117.81	3.39	.1153	NS
*D*	72.03	1	72.03	2.07	.2002	NS
*E*	0.0533	1	0.0533	0.0015	.9700	NS
Residual	208.67	6	34.78			
Corrected total	7306.78	11				
*R* ^2^	.9714					
Predicted *R* ^2^	.8858					
Adjusted *R* ^2^	.9476					
C.V. %	12.79					

^a^ *A*: extraction pH, *B*: NaCl concentration (M), *C*: extraction temperature (°C), *D*: liquid/solid ratio (mL g^−1^), *E*: extraction time (h).

^b^ Degree of freedom, S: Significant (*p* < .05); NS: Not significant (*p* > .05).

**FIGURE 1:**
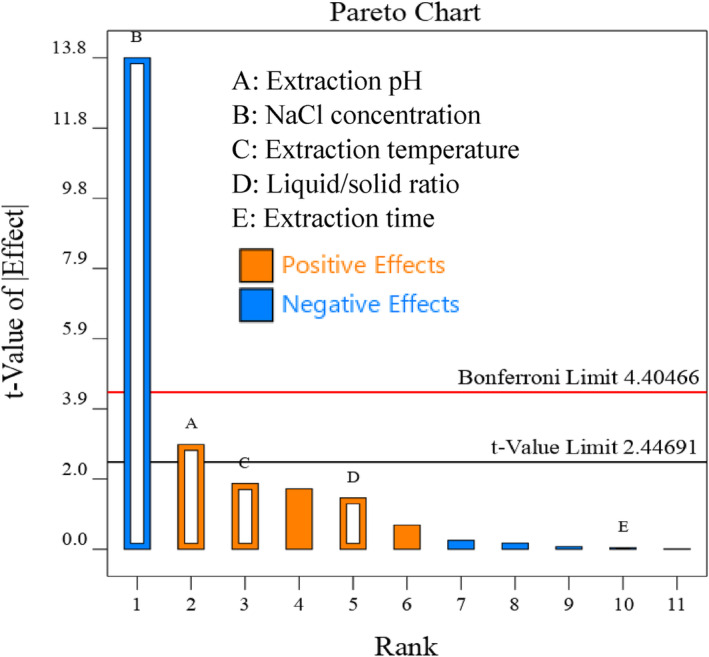
Illustration of Pareto chart from the Plackett–Burman design for the effects of independent variables on the protein extraction from pomegranate seeds.

The significance of the tested parameters as well as their positive or negative effects on the response variables, are illustrated in Figure 1 as a Pareto chart. The influence of each parameter on the response is proportional to the length of its respective bar. Bars lying above the *t*‐value limit are considered significant (*p* < .05) (Boateng & Yang, 2021). Among the parameters examined, liquid/solid ratio, temperature, and time did not significantly influence the protein recovery. This suggests that, within the studied range, these factors do not notably affect the solubility and, consequently, the extractability of PSP. The effects of these parameters on the extractability of plant proteins can vary based on both the extraction conditions and the source of protein. Cheng et al. (2021) found that extraction temperature did not significantly impact the extraction yield of protein from *Moringa oleifera* leaves. Also, Devi and Badwaik (2022) reported that extraction time within the range they studied (5–25 min) did not have a significant impact on the yield of protein extraction from muskmelon seed meal. However, Garg et al. (2020) found that both extraction temperature and time are among the main parameters influencing protein recovery from sangria (*Prosopis cineraria*) seed. In the current study, pH demonstrated a positive impact, whereas NaCl concentration exhibited a negative influence on the recovery of protein extraction. The findings of previous studies suggest that pH and NaCl concentration can influence the extractability of proteins by affecting their solubility levels and surface charges (Baca‐Bocanegra et al., 2021; Wang et al., 2020). Therefore, NaCl concentration and pH were chosen as the variables for further optimization of the extraction process in the current study. So far, no studies have evaluated the influence of such variables on the recovery of protein from pomegranate seeds. However, in agreement with the findings of the current work, studies on several plant proteins from different sources such as sunflower meal (Slabi et al., 2020), canola meal (Gerzhova et al., 2016), and hemp seed (Potin et al., 2019) revealed that NaCl concentration and pH were two important parameters significantly influencing the protein extraction from the studied sources.

### 3.2 Optimization of the extraction conditions for PSP

RSM with two‐factor‐three‐level FCCD was employed to identify the optimal values of the significant variables (pH and NaCl concentration) affecting PSP extraction. The observed responses corresponding to the FCCD experiments are given in Table 2. The analysis of experimental data via regression resulted in a polynomial model for the protein yield (*Y*) as follows:

(12) $$Y=43.81+8.03A-28.72B+1.03\mathrm{AB}+0.17A^{2}+6.63B^{2}$$

where *A* and *B* represent the coded values of pH and NaCl concentration, respectively. The ANOVA obtained for these data (Table 4) indicates that the model is significant (*p* < .0001). The correlation coefficient (*R* ^2^) was found to be .9743, which indicates the adequacy of the fitted model. Additionally, the adjusted *R* ^2^ and predicted *R* ^2^ values of .9559 and .7959, respectively, further support the model's adequacy. A low value of C.V. (9.69%) illustrated that the model is reproducible. The lack of fit for the model was also determined to be not significant (*p* > .05), indicating the validity of the model. The linear term NaCl concentration (*B*) and the linear term pH (*A*) are, respectively, the most and the second most effective parameters influencing protein extraction from pomegranate seed, and the quadratic term of NaCl concentration (*B* ^2^) is the third significant term (Table 4). However, the quadratic term of pH (*A* ^2^) as well as the interaction term of NaCl concentration and pH (AB) were determined to be not significant (*p* > .05). Therefore, the following simplified polynomial model is obtained after the elimination of non‐significant (*p* > .05) terms from the original model:

(13) $$Y=26.65+3.49A–104.43B+26.50B^{2}$$

where *Y*, *A*, and *B* represent the predicted value of protein yield, the actual values of pH, and NaCl concentration, respectively.

**TABLE 4:** Analysis of variance (ANOVA) for the yields of protein extraction from pomegranate seeds using face‐centered central composite design.

Source^a^	Sum of squares	*df* ^b^	Mean square	*F*‐value	*p*‐Value	Significance
Model	5484.10	5	1096.82	53.00	<.0001	S
*A*	387.21	1	387.21	18.71	.0035	S
*B*	4947.88	1	4947.88	239.07	<.0001	S
*AB*	4.20	1	4.20	0.2031	.6659	NS
*A* ^2^	0.0854	1	0.0854	0.0041	.9506	NS
*B* ^2^	121.25	1	121.25	5.86	.0461	S
Residual	144.88	7	20.70			
Lack of fit	108.36	3	36.12	3.96	.1086	NS
Pure error	36.51	4	9.13			
Corrected total	5628.97	12				
*R* ^2^	.9743					
Predicted *R* ^2^	.7959					
Adjusted *R* ^2^	.9559					
C.V. (%)	9.69					

^a^ *A*: Extraction pH, *B*: NaCl concentration (M).

^b^ Degree of freedom, S: Significant (*p* < .05); NS: Not significant (*p* > .05).

**FIGURE 2:**
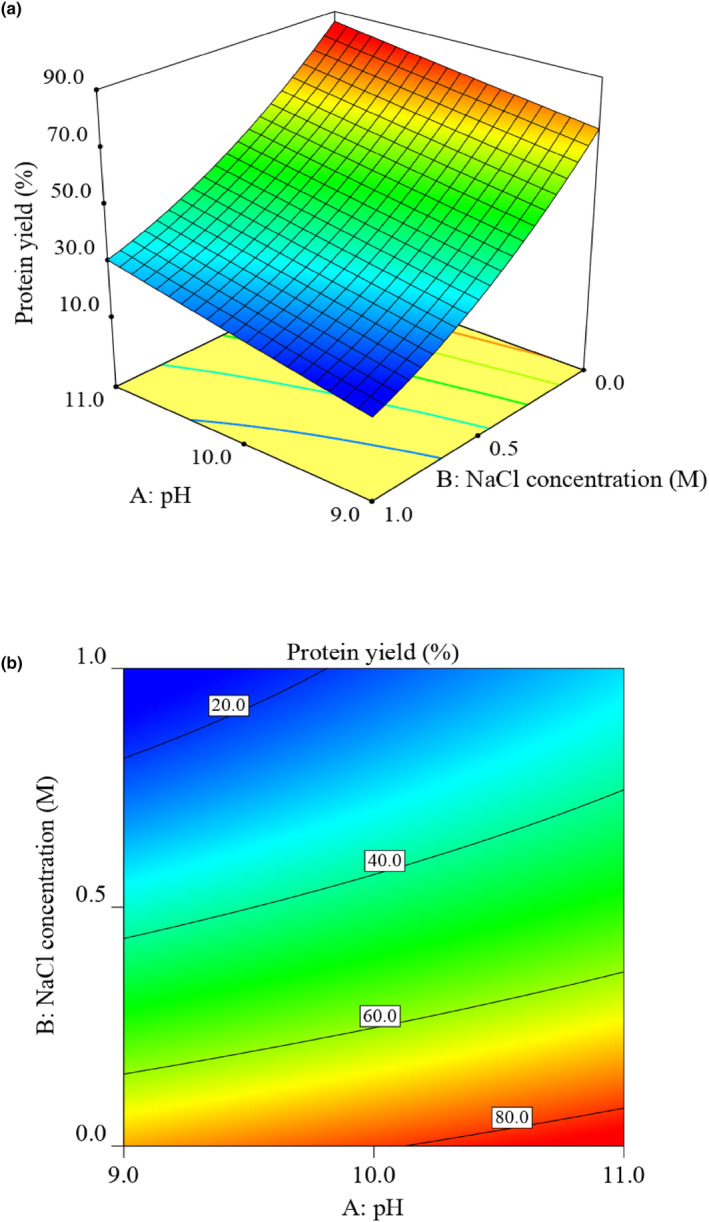
Three‐dimensional plot (a) and contour plot (b) showing the effects of pH and NaCl concentration on the yield of protein extraction from pomegranate seeds. Other extraction parameters were maintained at the following values: extraction time, 1 h; extraction temperature, 25°C; liquid/solid ratio, 20:1 mL mg^−1^.

Figure 2 shows the effects of the two significant parameters (pH and NaCl concentration) on the PSP extraction yield. At a fixed NaCl concentration, the yield of protein extraction increased as pH rose from 9.0 to 11.0. This observation indicates that strong alkaline conditions favor protein recovery from pomegranate seeds. Alkaline conditions can increase the surface charge in protein molecules and consequently facilitate protein solubility in water. This is due to the ionization of acidic and neutral amino acids in alkaline conditions, which results in increased protein recovery during the extraction process (Kumar et al., 2021). Similar to this finding, Guerreo‐Ochoa et al. (2015) reported that maximum yield of protein extraction from quinoa seeds was obtained at pH 11.0. Orellana‐Palacios et al. (2022) also reported that maximum recovery of protein from cherimoya seed was achieved at pH 10.5. Bernardi et al. (2018) optimized protein extraction from rice bran using an alkaline extraction technique and noted that the highest protein recovery was achieved at pH 10. Alkaline conditions not only allow for a high recovery of protein from plant sources but also enhance its bioavailability and digestibility (Kumar et al., 2021). Based on three‐dimensional (Figure 2a) and contour (Figure 2b) plots, a considerable decline in the extraction yield is evident with an increase in the NaCl concentration at a given pH level. Therefore, it is best not to use NaCl for protein extraction from pomegranate seeds. Similar findings on protein extraction from quinoa seeds were reported by Guerreo‐Ochoa et al. (2015). Conversely, several other studies have reported that low concentrations of NaCl (0.0–1.0 M) can lead to higher protein extraction from several plant sources such as rapeseed meal (Fetzer et al., 2018), canola meal (Gerzhova et al., 2016), and hemp seed (Potin et al., 2019). According to Wang et al. (2020), the extractability of proteins may be influenced by their hydrophobicity values and surface properties. In the current study, the decrease in protein extraction observed upon the addition of NaCl at 0.5‐ and 1.0‐M levels could be attributed to the salting‐out effect of NaCl on the solubility of PSP, which is explained by the weakened protein‐water interactions as a result of binding of the ions with water molecules (Gerzhova et al., 2016). Nevertheless, it is important to consider that the effect of NaCl addition on protein extraction can vary depending on the pH of the extraction medium. This variability may arise from the conformational changes of protein molecules at different pH levels and also their interactions with different types of compounds present in the meal (Potin et al., 2019). Although both pH and NaCl concentration demonstrated significant effects on the yield of protein extraction, their interaction was found to be not significant (Table 4). Accordingly, the predicted conditions to achieve the highest protein extraction were obtained as follows: extraction pH at 11.0 and NaCl concentration at 0.0 M. The yield obtained under the experimental conditions (83.8 ± 2.6%) was not significantly different from the value predicted by RSM for the optimum conditions (86.3%). Therefore, it can be concluded that the current RSM model for the extraction of PSP is valid and reliable.

### 3.3 Amino acid composition of PSPI

The amino acid profile of PSPI, as detailed in Table 5, revealed glutamic acid as the most abundant, contributing 26.8 g per 100 g^−1^ of protein. Additionally, leucine was identified as the second most abundant amino acid at 9.1 g per 100 g^−1^ of protein. Glutamic acid is known for its antioxidant properties (Deng et al., 2020), while leucine is known for its role in preventing muscle breakdown and enhancing the glycemic index in type 2 diabetic patients (Yang et al., 2010).

**TABLE 5:** Amino acid composition of pomegranate seed protein isolate (PSPI) as compared to that recommended by FAO/WHO/UNU for adults.

Amino acid	PSPI	FAO/WHO^a^
Essential amino acids		
Histidine	4.6 ± 0.2	1.5
Leucine	9.1 ± 0.4	5.9
Isoleucine	1.6 ± 0.1	3.0
Lysine	5.4 ± 0.6	4.5
Methionine	2.3 ± 0.1	2.2^b^
Phenylalanine	5.0 ± 0.3	3.0^c^
Threonine	4.2 ± 0.4	2.3
Valine	3.6 ± 0.1	3.9
Non‐essential amino acids		
Serine	4.4 ± 0.1	
Glycine	3.1 ± 1.9	
Tyrosine	3.2 ± 0.1	
Arginine	7.7 ± 0.3	
Alanine	3.9 ± 0.1	
Proline	8.3 ± 0.1	
Aspartic acid	6.9 ± 0.1	
Glutamic acid	26.8 ± 0.0	
Amino acid distribution		
Hydrophilic^d^	11.7 ± 0.2	
Hydrophobic^e^	36.8 ± 0.7	
Acidic^f^	33.7 ± 0.1	
Basic^g^	17.7 ± 1.1	
E/T (%)^h^	35.7	

*Note*: All measurements were carried out in triplicates. The results are reported as g amino acid per 100 g protein.

^a^ Essential amino acid requirement reported by WHO/FAO/UNU (2007) for adults.

^b^ Methionine + cysteine.

^c^ Phenylalanine + tyrosine.

^d^ Hydrophilic amino acids: threonine, serine, cysteine, tyrosine.

^e^ Hydrophobic amino acids: glycine, proline, alanine, leucine, valine, methionine, phenylalanine, isoleucine.

^f^ Acidic amino acids: glutamic acid, aspartic acid.

^g^ Basic amino acids: lysine, histidine, arginine.

^h^ E/T: Ratio of essential amino acids to total amino acids.

Among the amino acid categories (Table 5), the hydrophobic amino acids were found at a higher level (36.8 g 100 g^−1^ protein) compared to the acidic amino acids (33.7 g 100 g^−1^ protein), basic amino acids (17.7 g 100 g^−1^ protein) and hydrophilic amino acids (11.7 g 100 g^−1^ protein). According to Deng et al. (2020), hydrophobic amino acids have significant roles in improving the thermostability levels of proteins by forming a compact core in the inner part of the protein molecule. Also, the E/T (essential to total amino acids) ratio in PSPI (35.7%) exceeded that reported for soy protein isolate (32.7%) in the study by Feyzi et al. (2015). This finding implies that PSPI has a high potential to replace soy protein isolate as an emerging plant protein source.

To gain an insight into the nutritional properties of PSPI, its essential amino acids were compared against the recommended levels from WHO/FAO/UNU (2007) for adults (Table 5). The levels of essential amino acids (except for valine and isoleucine) in PSPI exceeded the recommended levels by WHO/FAO/UNU (2007) for adults. Therefore, PSPI can be a good source of protein for adults if slight amounts of isoleucine and valine are also added to the formulation.

### 3.4 Thermal properties of PSPI

Thermal properties of PSPI are presented in Table 6. PSPI denaturation temperature (*T*_*d*_), which was observed as an endothermic peak in the PSPI thermogram, was equal to 89.5°C. Amino acid profile and protein structure are the primary factors that determine the *T*_*d*_ of proteins (Mir et al., 2019). In addition, hydrophobic interactions improve the thermal stability of proteins and result in higher *T*_*d*_ values (Deng et al., 2019). The onset and end set temperatures were found at 44.5 and 187.8°C, respectively. The required energy for protein denaturation, referred to as enthalpy (∆*H*), was at 11.5 J g^−1^. The denaturation temperature of PSPI was in line with those of various protein isolates reported in the literature, which shows that PSPI has good thermal stability when used in food systems. Heating leads to the breakdown of the bonds and unfolding of the proteins leading to an endothermic peak in the thermogram of proteins (Gundogan & Karaca, 2020).

**TABLE 6:** Thermal and functional properties of the extracted PSPI.

Parameters	PSPI
Thermal properties	
*T*_0_ (°C)	44.5 ± 0.2
*T*_*d*_ (°C)	89.5 ± 0.7
*T*_*e*_ (°C)	187.8 ± 0.5
∆*H* (J g^−1^)	11.5 ± 0.9
Functional properties	
WHC (g g^−1^)	3.7 ± 0.0
OHC (g g^−1^)	4.3 ± 0.0
In vitro digestibility (%, w/w)	74.3 ± 1.2

*Note*: All measurements were carried out in triplicates.

Abbreviations: ∆*H*, Enthalpy; OHC, Oil‐holding capacity; *T*_0_, Onset temperature; *T*_*d*_, Denaturation temperature; *T*_*e*_, End set temperature; WHC, Water‐holding capacity.

### 3.5 Functional properties of PSPI

#### 3.5.1 Protein solubility profile

**FIGURE 3:**
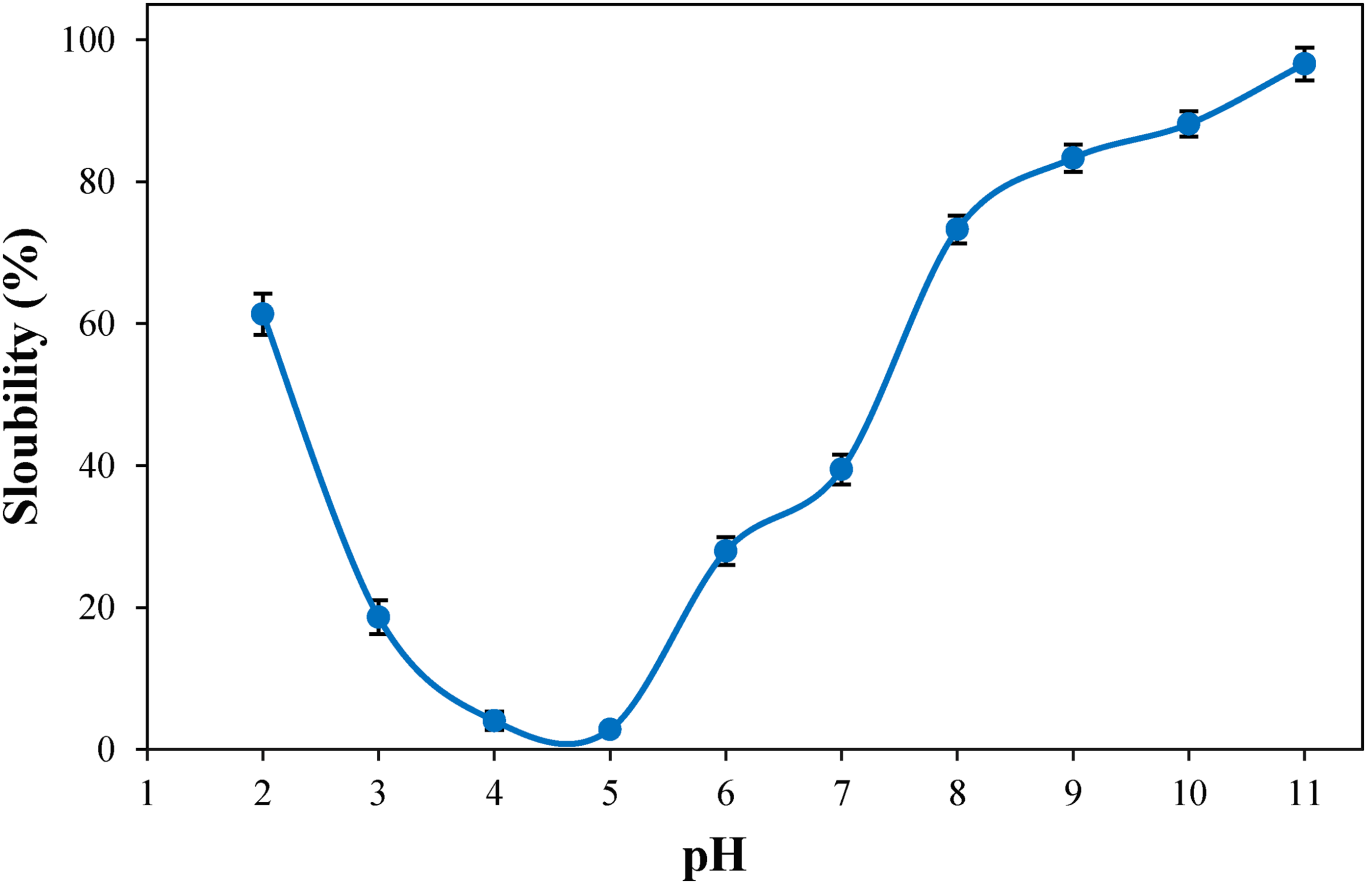
Changes in the solubility of PSPI with pH. All measurements were carried out in triplicates.

Solubility stands as a crucial functional characteristic of proteins as it can influence their capabilities in forming gels, emulsions, and foams (Perović et al., 2020). Figure 3 displays the solubility profile of PSPI. The minimum solubility of PSPI was observed near the isoelectric pH (4.0–5.0). Near the isoelectric pH, the equilibrium between positive and negative charges can diminish electrostatic repulsion between protein molecules, resulting in decreased solubility and precipitation. The solubility of PSPI was increased as pH deviated from the isoelectric point. This phenomenon arises from the increased interactions between proteins and water at pH levels both above and below the isoelectric point (Hadidi et al., 2023). At pH 7.0, the protein solubility of PSPI (39.5%) closely resembled that of soybean protein isolate (40.8%) (Hu et al., 2010). Furthermore, maximum solubility for PSPI (96.6%) was obtained at pH 11.0, which was higher than that observed for other plant proteins such as soybean (86.7%) (Han et al., 2024), quince seed (87.5%) (Deng et al., 2019) and walnut (86.8%) (Ma et al., 2024) protein isolates. Differences in the solubility levels of proteins might be linked to variations in their amino acid composition and surface hydrophobicity.

#### 3.5.2 Water‐holding capacity

WHC of PSPI was determined to be 3.7 g g^−1^ (Table 6), which is higher than those of a variety of pulse protein concentrates, ranging from 1.4 to 2.3 g g^−1^ (Ruckmangathan et al., 2022). The ability of proteins to maintain water in their three‐dimensional structures is defined as WHC. Conformational characteristics, the ratio of surface polarity to hydrophobicity, amino acid profile, and particle size are the key parameters that determine the WHC of proteins (Dabbour et al., 2018). The WHC of a protein is an important parameter influencing the texture, viscosity, and mouthfeel of the ultimate food product. Typically, the recommended WHC value for the proteins used as ingredients in viscous foods (such as baked doughs, pasta, and soups) is between 1.49 and 4.72 g g^−1^ (Aletor et al., 2002). Therefore, due to having favorable WHC, PSPI can be considered for application in such food products.

#### 3.5.3 Oil‐holding capacity

The OHC obtained for PSPI in this study was 4.3 g g^−1^ (Table 6), which is significantly higher than those of protein isolates from various pulses (ranging from 0.86 to 1.43 g g^−1^) (Ma et al., 2022) and oilseeds (ranging from 0.86 to 1.32 g g^−1^) (Gao et al., 2021). Furthermore, this value was found to be higher than those published by Talekar et al. (2018) for raw pomegranate seed powder (0.77 g g^−1^) and pomegranate seed residue remaining after oil extraction (2.3 g g^−1^). OHC levels in proteins, which reflect their ability levels to absorb oil, have a significant influence on the shelf‐life and flavor retention of foods (Dabbour et al., 2018). The key factors influencing the OHC of proteins include the type and amount of protein, surface hydrophobicity, and the oil used (Deng et al., 2019). A high OHC value (4.3 g g^−1^) for PSPI is an asset for using this protein isolate in such products as sausages, cake batters, and sauces with a high‐fat content.

#### 3.5.4 Emulsion properties

**FIGURE 4:**
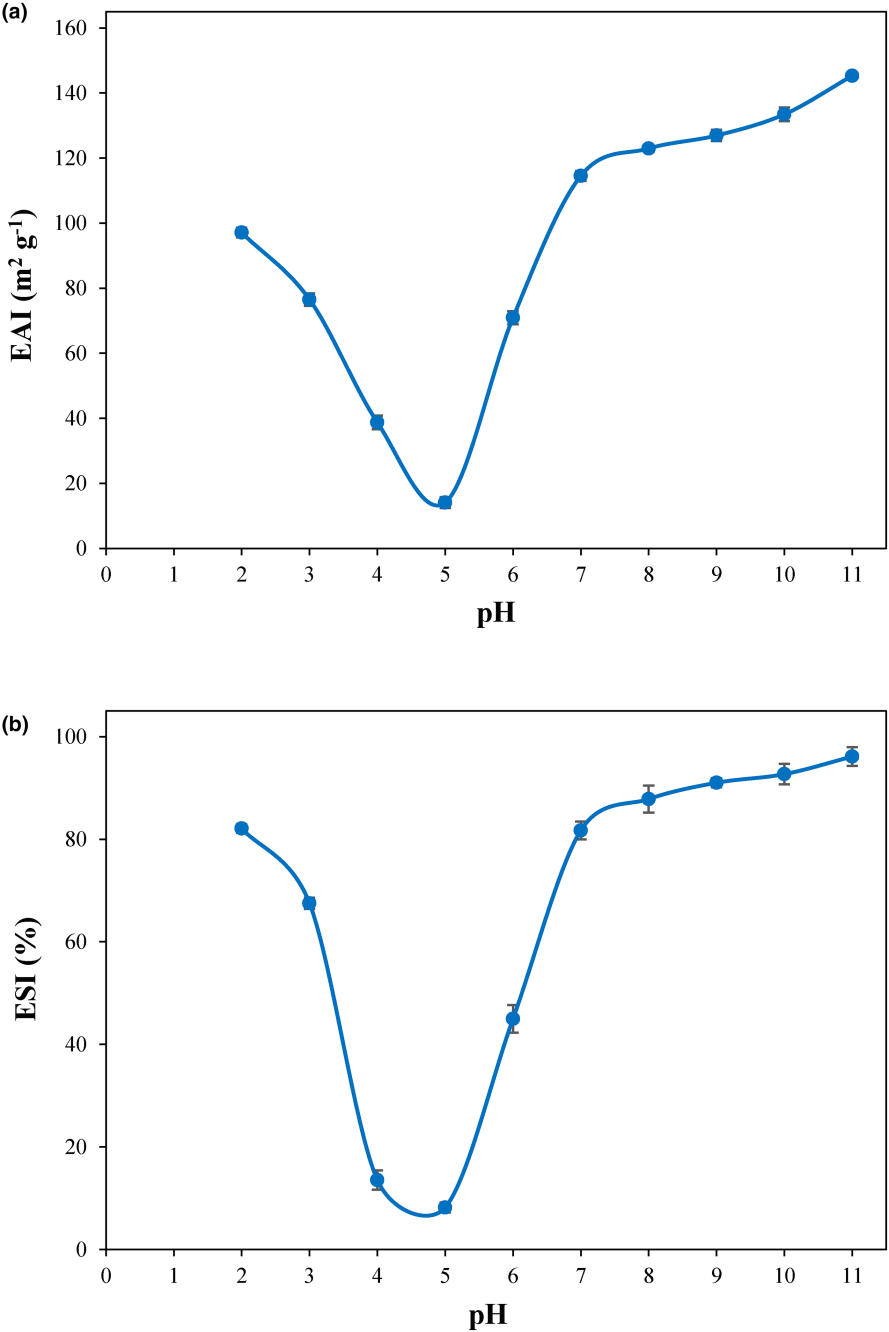
Changes in the emulsifying activity index (EAI) (a) and emulsion stability index (ESI) (b) of PSPI with pH. All measurements were carried out in triplicates.

EAI and ESI for PSPI were assessed at various pH levels over 2.0 to 11.0 (Figure 4a,b). EAI refers to the protein's ability to form an emulsion (Kaushik et al., 2016). After an emulsion is formed, its stability is evaluated using a different parameter, so‐called ESI (Kaushik et al., 2016). Changes in both EAI and ESI with a change in pH level were similar to those of the solubility (U‐shaped curves) and net surface charge. Higher EAI and ESI were observed in extremely alkaline and acidic environments, where higher solubility and net surface charge were also seen. At the isoelectric pH, the EAI was measured at its lowest (14.1 m^2^ g^−1^), along with ESI at 8.18%, correlating with the observed solubility profile of PSPI. Poor solubility at the isoelectric pH decreases the number of dissolved protein molecules; therefore, fewer protein molecules interact at the interface of oil and water (Sahni et al., 2020). Net surface charge is another important factor that is in the lowest level at the isoelectric pH where more protein molecules aggregate and precipitate and cannot unfold at the interface of oil and water (Deng et al., 2019). A good balance between hydrophobic and hydrophilic amino acids of the protein can enhance its emulsifying properties in a way that protein molecules will disperse into the interface of water and oil more quickly, and their conformations will be changed to adsorb higher levels of oil (Gundogan & Karaca, 2020).

#### 3.5.5 Foaming properties

**FIGURE 5:**
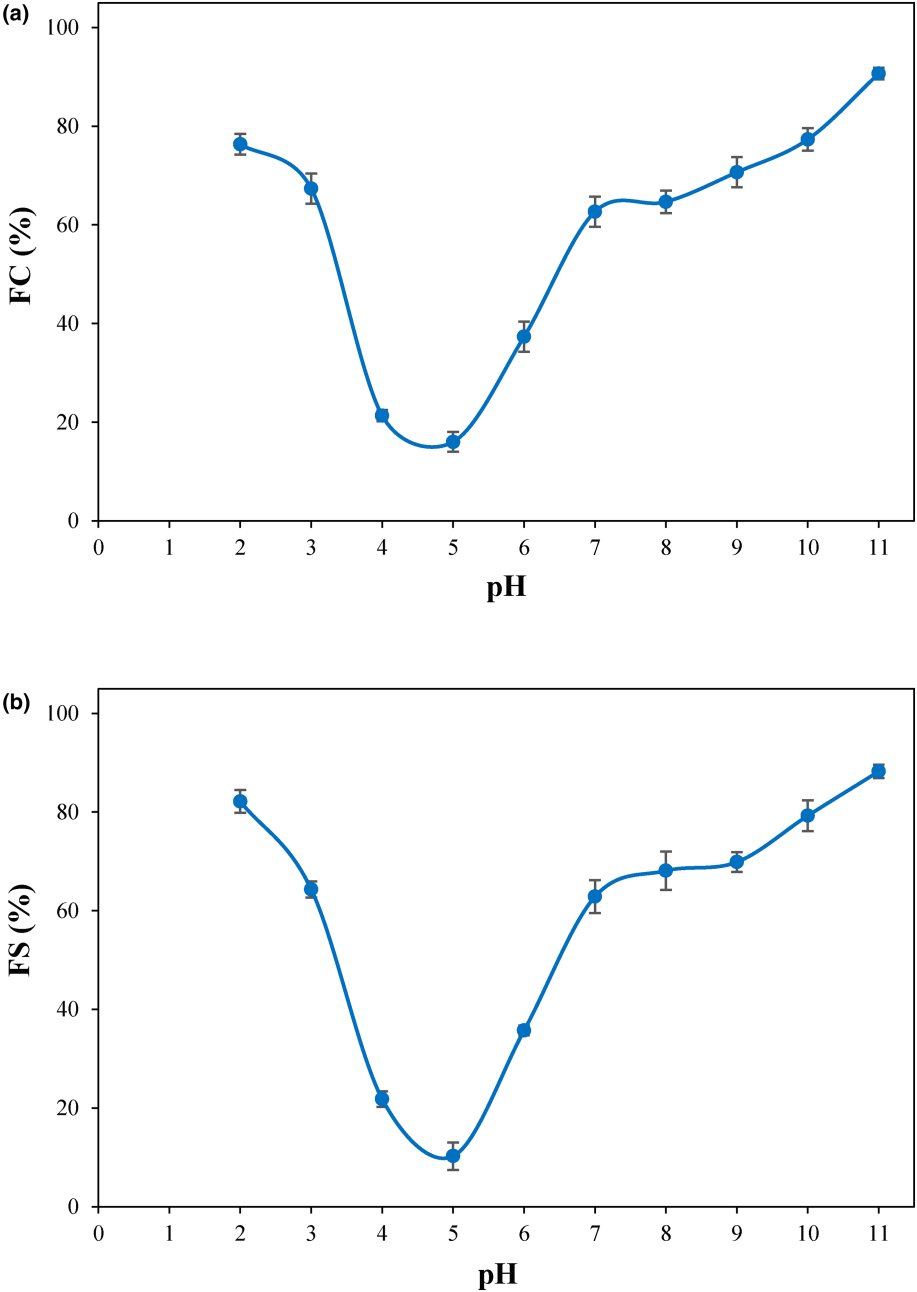
Changes in the foaming capacity (FC) (a) and foam stability (FS) (b) of PSPI with pH. All measurements were carried out in triplicates.

Figure 5a,b shows the FC and FS values of PSPI at different pH levels. The lowest FC and FS values (16.0% and 10.2%, respectively) were found at pH 5.0 (the isoelectric pH of PSPI), which can be correlated with the minimum solubility levels as well as zero surface charges of proteins at the isoelectric pH. Higher levels of solubility and surface charge result in higher FC and FS. Foaming is considered an important property for proteins to be used in food systems. Therefore, the formation of high‐volume and stable foams can expand their utilization in the food industry, especially in whipped cream and ice cream mixes and in baked food products where overrun and aeration are desirable (Shevkani et al., 2015). A fine balance between hydrophobic and hydrophilic amino acids will also lead to good foaming (Gundogan & Karaca, 2020).

A lack of repulsive forces among protein molecules at the isoelectric pH will lead to their aggregation and consequently the coalescence of air bubbles will be increased (Shen et al., 2021). In acidic or alkaline conditions, PSPI exhibited increased foaming properties due to its elevated protein solubility. This phenomenon arises from the elevation in the net charge of the proteins in an aqueous solution and consequently the reduction in hydrophobic interactions and an enhancement in protein solubility. The enhanced solubility also helps maintain the air bubbles in the product leading to more stable foams at acidic and alkaline conditions (Deng et al., 2019).

### 3.6 In vitro digestibility

PSPI exhibited an in vitro protein digestibility of 74.3% (Table 6). Although this value is lower than those of such reference proteins as soy protein isolate, its digestibility level is still acceptable as an ingredient for application in food products. Protein digestibility is an important criterion in determining protein nutritional value. Type of enzyme, protein conformation, digestion model, time, presence of impurities such as anti‐nutrients as well as the method used for the extraction of the protein can influence the in vitro digestibility of proteins (Nasrabadi et al., 2021; Ruckmangathan et al., 2022; Talekar et al., 2018; Yu et al., 2017).

## 4 CONCLUSIONS

In the current study, the conditions for protein extraction from pomegranate seeds were optimized. PBD data showed that pH and NaCl concentration significantly influence PSP extraction. In the next step, FCCD was used for the optimization of these variables. A protein yield of 83.8% was obtained under the optimum conditions (pH 11.0 and NaCl concentration of 0.0 M). PSPI with 92.4 g per 100 g protein content was prepared by the isoelectric precipitation of PSP extracted under the optimized conditions. PSPI was found to be rich in key essential amino acids. It also exhibited favorable WHC, OHC, emulsifying, and foaming properties, particularly under alkaline conditions. PSPI also showed a high denaturation temperature, making it a suitable ingredient for use in foods subjected to heat treatment. The results of in vitro digestibility of PSPI showed that proteins of pomegranate seed have a relatively high digestibility. In summary of the current study's findings, PSPI can be considered as a novel protein source to be investigated further for utilization in numerous food formulations, especially in viscous foods and high‐fat food products.

## AUTHOR CONTRIBUTIONS

**Souri Oroumei:** Data curation (equal); formal analysis (equal); methodology (equal); software (equal); writing – original draft (equal). **Karamatollah Rezaei:** Conceptualization (equal); project administration (equal); resources (equal); supervision (equal); validation (equal); writing – review and editing (equal). **Hooman Chodar Moghadas:** Formal analysis (equal); investigation (equal); methodology (equal); software (equal); writing – original draft (equal).

## CONFLICT OF INTEREST STATEMENT

The authors declare no conflicts of interest.

## ETHICAL APPROVAL

This study does not involve any human or animal testing.

## Data Availability

Data will be available upon request.
